# Feasibility of a 2-minute eye-tracking protocol to support the early identification of autism

**DOI:** 10.1038/s41598-024-55643-z

**Published:** 2024-03-01

**Authors:** Lacey Chetcuti, Kandice J. Varcin, Maryam Boutrus, Jodie Smith, Catherine A. Bent, Andrew J. O. Whitehouse, Kristelle Hudry

**Affiliations:** 1 Department of Psychology, Counselling and Therapy, School of Psychology and Public Health, La Trobe University, Melbourne, VIC Australia; 2 Menzies Health Institute Queensland, Griffith University, Gold Coast, Australia; 3 Telethon Kids Institute, University of Western Australia, Perth, Australia; 4 School of Allied Health, Human Services and Sport, La Trobe University, Melbourne, VIC Australia

**Keywords:** Disability, Risk factors

## Abstract

We tested the potential for *Gazefinder* eye-tracking to support early autism identification, including feasible use with infants, and preliminary concurrent validity of trial-level gaze data against clinical assessment scores. We embedded the ~ 2-min ‘Scene 1S4’ protocol within a comprehensive clinical assessment for 54 consecutively-referred, clinically-indicated infants (prematurity-corrected age 9–14 months). Alongside % tracking rate as a broad indicator of feasible assessment/data capture, we report infant gaze data to pre-specified regions of interest (ROI) across four trial types and associations with scores on established clinical/behavioural tools. Most infants tolerated *Gazefinder* eye-tracking well, returning high overall % tracking rate. As a group, infants directed more gaze towards social vs. non-social (or more vs. less socially-salient) ROIs within trials. Behavioural autism features were correlated with increased gaze towards non-social/geometry (vs. social/people) scenes. No associations were found for gaze directed to ROIs within other stimulus types. Notably, there were no associations between developmental/cognitive ability or adaptive behaviour with gaze towards any ROI. *Gazefinder* assessment seems highly feasible with clinically-indicated infants, and the people vs. geometry stimuli show concurrent predictive validity for behavioural autism features. Aggregating data *across* the ~ 2-min autism identification protocol might plausibly offer greater utility than stimulus-level analysis alone.

## Introduction

Autism spectrum disorder (hereafter, *autism*) is diagnosed when behavioural impairments converge across two broad functional domains: social-communication and behavioural flexibility^1^. Individual presentation is otherwise highly heterogeneous^2^ and identification and diagnosis rely on clinical judgement of behavioural presentation, typically through direct observation and informant report^3^. This process can be protracted, bringing substantial delays to young autistic children and their families accessing critical therapeutic supports. Hence, there is an identified need for more accurate and efficient assessment processes, particularly in identifying emerging signs of autism during infancy^4^ to facilitate supports while the developing brain is highly plastic, and before possible associated disability becomes entrenched^5^.

Technology offers the potential to improve diagnostic accuracy and efficiency by reducing the current reliance on the experience and confidence of individual clinical professionals^6,7^. Technology also has the capacity to support the earlier identification of autism through biomarker discovery^8^. Eye-tracking is one promising such approach, offering objective insights into the emergence and consolidation of autism in early childhood. Indeed, the application of eye-tracking in research programs and autism clinics has been steadily increasing^9^.

### Eye-tracking as potential autism identification tool

The potential for eye-tracking to contribute to the earlier identification and diagnosis of autism is grounded in well-documented visual attention differences between people with and without autism. In a seminal meta-analysis, Frazier et al.^10^ quantified the magnitude and significance of autism-related gaze differences, aggregating data across 122 independent case–control studies to show robust gaze differences across a wide range of stimulus types and specific within-stimulus regions of interest (ROIs). Notably, there were marked differences between autistic and non-autistic individuals in gaze towards non-social vs. social ROIs, and towards eye- and whole-face ROIs compared to nose-, mouth-, and body ROIs within human/interactive stimuli. Findings from another, more recent meta-analysis of 22 eye-tracking studies similarly concluded that gaze towards eye-regions of the human face and towards non-social scene ROIs effectively discriminated adult viewers with and without autism^11^.

Adjacent literature has evaluated the clinical utility of eye-tracking based visual social attention measurement to support autism diagnosis, including among young children deemed to be at increased likelihood due to family history of autism or the presentation of suggestive clinical/behavioural indicators (e.g.,^12,13^). Here, studies have provided complimentary evidence of increased number or duration of gaze fixations to non-social vs. social scenes (e.g., objects or geometric images vs. people^14^) and to typically less socially-salient elements within social scenes (e.g., people and faces) among 6-month-olds who later received an autism diagnosis^15^. Indeed, the performance properties of eye-tracking data for discriminating young children who do vs. do not subsequently receive an autism diagnosis is high, with sensitivity and specificity estimates similar to those from behavioural assessments historically adopted to support clinical decision-making^16^.

Despite the demonstrated potential of eye-tracking to discriminate individuals with and without autism, recent reviews have noted inconsistencies in individual study findings, partly attributable to methodological differences including features of the stimuli presented for viewing^10,13^. An important consideration towards achieving clinically-useful eye-tracking assessment is for standardized, validated and scalable paradigms. An approach whereby gaze data are gathered consistently—with standard apparatus, stimulus presentation, and pre-determined ROIs for gaze data capture—across samples from a given population of interest, might better support efforts to achieve an objective autism identification and diagnostic support tool, with stronger inferences drawn from a pool of studies founded on a common approach.

Additionally, to be clinically useful, eye-tracking technology will need to be feasibly incorporated within clinical care contexts. A promising approach will only be useful if the protocol is well-tolerated by its intended viewers—returning sufficient data to afford confident conclusions—and operable and interpretable by its intended users. In the context of seeking to support the earlier identification and diagnosis of autism, objective technology-based tools will only be useful if engaging for infant viewers and easily operable by clinical personnel with minimal technical training.

### The brief, standardised, and scalable Gazefinder protocol

Designed for ‘off the shelf’ use, JVCKENWOOD Corporation’s (JKC) *Gazefinder* eye-tracking technology (GP-100EA, detailed in Supplementary Figures S3–S6) comprises hardware and a standardized brief audio-visual presentation (referred to as ‘Scene 1S4’; Supplementary Figure S7, Tables S2–S3) featuring a collection of key stimuli previously used in autism eye-tracking research—five static and animated human face trials, six animated paired social and non-social scenes (i.e., people vs. geometry in various configurations), two biological motion PLDs animations, and two animated referential attention probes. Pre-specified ROIs (as detailed in Supplementary Figure S7, Tables S2–S3) allow for automatic visualisation of a viewer’s gaze data after assessment and ready exportation from the device for further in-depth analysis.

Previous research conducted independently from our team indicate that the *Gazefinder* ‘Scene 1S4’ animation effectively differentiates individuals with and without autism in childhood and adolescence/adulthood, with estimated sensitivity and specificity > 74% in childhood (5–17 years)^17^ and > 80% in adolescence/adulthood^18^. Recent evidence further supports the feasibility of using *Gazefinder* with infants sampled from the general population, showing face validity with greater group mean-level gaze towards more vs. less socially-salient ROIs^19^.

### The current study

Extending investigation into the potential for *Gazefinder* as a standardised, scalable autism evaluation tool, we explored whether this technology might be feasibly incorporated into an assessment schedule around the time when first signs of autism often come to the attention of caregivers and professionals. Specifically, we tested the feasibility of using *Gazefinder* with a heterogeneous clinically-indicated sample of infants, enrolled into a larger research program due to identified early signs of autism. We incorporated *Gazefinder* GP-100EA devices with standardised ‘Scene 1S4’ autism assessment into appointments otherwise mirroring a clinical autism evaluation, for a proof-of-concept test of clinical implementation. Our two-fold focus was on: (1) the feasible incorporation of *Gazefinder* for quality data capture with infant viewers presenting with diverse clinical histories and presentation, and (2) the preliminary validity of gaze data to pre-specified ROIs evidenced through within-stimulus preferential attention and concurrent association with established clinical/behavioural assessments administered at the same appointment.

Regarding feasibility, we were interested in the successful incorporation of *Gazefinder* assessment within the broader clinical study protocol and tolerance by infant viewers. Specifically, we operationalised feasibility in terms of successful gaze detection during an initial calibration sequence, and the subsequent overall gaze data capture (i.e., % tracking rate) by *Gazefinder* across the ~ 2-min stimulus sequence.

Regarding preliminary validity, we hypothesised that infants as a group would show broad preferential attention towards social vs. non-social and more vs. less socially-salient ROIs within stimulus types: eyes (vs. mouth) of a human face (particularly in static presentations vs. animated scenes with mouth moving/talking), people (vs. geometry) in preferential attention pairs, upright (vs. inverted) biological motion point-light displays (PLD), and referential agent and target object (vs. distractors) in referential attention probes.

Further, we hypothesised that clinical assessment scores suggesting more signs of autism would be associated with more gaze towards non-social/less socially-salient ROIs, and less gaze towards social/more socially-salient ROIs. Given intended specificity of *Gazefinder’s* ‘Scene 1S4’ protocol to assess autism-related visual attention differences, we did not anticipate any significant associations between gaze data and clinical scores on broader developmental/cognitive ability or adaptive functioning assessments. However, given the important role of shared and referential (i.e., dyadic and triadic) attention for language development^20^, which is often delayed or different in autism^21^, we anticipated that infants with poorer assessed language skills might show less gaze towards agent/target during specific referential attention scenes of the ‘Scene 1S4’ autism assessment.

## Methods

### Design and procedure

This evaluation was embedded within a larger research program enrolling community-referred infants showing early signs of possible emerging autism^22,23^, with experimental tasks (including eye-tracking) incorporated alongside a clinical/behavioural assessment battery. This research was approved by the Child and Adolescent Health Service Ethics Committee (CAHSEC Ref# 2016008EP; June 8th 2016) and performed in accordance with the Declaration of Helsinki with each caregiver providing written informed consent for their infant’s participation at the start of the appointment. This broader research program offered an opportune context within which to examine the feasible use of *Gazefinder* within an assessment schedule otherwise mirroring activities incorporated in clinical early identification and assessment for autism, and involving a participant sample with diverse clinical family medical histories and personal presentations. As *Gazefinder* devices were available for incorporation into the larger research program around recruitment mid-point, data reported here are for a subgroup (52%) of the full recruited cohort. Specifically, participants contributing data here were all consecutively-enrolled infants seen from January 2017 to February 2018.

Inclusion criteria for infant participants were prematurity-adjusted age 9–14-months (i.e., corrected if gestational age < 37 weeks), and clinical-indication of increased autism likelihood, operationalised as ≥ 3 of 5 key early autism behaviours according to the Social Attention and Communication Surveillance–Revised (SACS-R) developmental surveillance tool 12-month check^24^. Potentially eligible infants were referred from community Maternal and Child Health Nurses trained to undertake SACS-R monitoring with infants attending their routine child health/development checks, or following file review for infants engaged with a community Child Development Service^22^. In both cases, formal eligibility against the SACS-R tool was confirmed through discussion with each infant’s parent/caregiver at the point of study enrolment. The in-person assessment was scheduled where possible, within the following 2 weeks. Additional specified inclusion/exclusion criteria for the research were sufficient parental English-language capacity for full study participation, absence of any pre-identified developmental/neurological condition in the infant (including substantially pre-term birth < 32 weeks’ gestational age), and no family intention to relocate within the planned 2-year follow-up time.

Eligible infants whose parents consented to their participation attended an appointment comprising direct- and parent-reported clinical/behavioural assessments and experimental tasks (including *Gazefinder* eye-tracking once apparatus were available in January 2017). The clinical researchers responsible for completing all assessments had no specific disciplinary expertise of/extensive experience using eye-tracking technology, and were provided with an operational manual and one-off demonstration of *Gazefinder* use by its manufacturers. Clinical/behavioural measures were administered and scored blind to infants’ eye-tracking data (i.e., all analysis of *Gazefinder* data occurred after the analysis and reporting of clinical/behavioural data^22^).

### Clinical/behavioural phenotyping

The clinical/behavioural assessment protocol included the semi-structured, play-based Autism Observation Scale for Infants (AOSI)^25^ offering a measure of emerging autism signs. Administration was by trained clinical researchers who showed high intra- and inter-rater agreement^22,26^. Higher AOSI total scores signal greater autism-related behaviours, with scores ≥ 9 suggestive of high likelihood of a later autism diagnosis^27,28^.

The clinical/behavioural protocol also included two standardised, norm-referenced measures of broader developmental/cognitive abilities, and adaptive behaviours that regularly feature in clinical autism research^21,27,29^. The Mullen Scales of Early Learning (MSEL) indexes overall developmental/cognitive abilities with excellent test–retest and inter-scorer reliability for infants aged ≤ 24-months (*r* ≥ 0.82)^30^. A summary Non-Verbal Developmental Quotient (NVDQ) was derived for analysis by averaging infants’ age-equivalence (AE) scores across Visual Reception and Fine Motor scales, dividing by infant chronological age (prematurity adjusted where relevant), and then multiplying the result by 100. Additionally, MSEL Receptive Language and Expressive Language scale AE scores were retained separately for analysis of specific association with emerging language skills. The Vineland Adaptive Behavior Scales-2nd Edition (VABS-II) is a caregiver-report adaptive behaviour measure, showing excellent test–retest and inter-rater reliability for infants aged ≤ 24-months (*r* ≥ 0.81)^31^. The VABS-II Adaptive Behavior Composite (ABC) Standard Scores (SS; population *M* = 100, *SD* = 15) was retained for analysis, which summarises functioning across Communication, Daily Living (i.e., personal independence), Socialization, and Motor skill domains.

Also of relevance within the larger trial protocol was the 191-item, caregiver-reported Infant Behavior Questionnaire-Revised (IBQ-R)^32^ which quantifies temperament traits across three domains potentially informative of *Gazefinder* tolerability: activity level, distress to limitations, and duration of orienting. Drawing on data from the larger cohort, we have previously shown internal consistency reliability across the IBQ-R, and predictive validity of domain scores for later child behaviour problems^33^.

### Eye-tracking protocol

*Gazefinder* model GP-100 EA was used for the current study, with product specifications detailed in manufacturer Instruction Manual^34,35^ and key details from these included in Supplementary Figures S3–6. This *Gazefinder* apparatus includes a 19-inch monitor (1280 × 1024 pixels) with integrated infrared light and camera to capture viewers’ corneal reflection of eye position at 50 Hz (i.e., 3000 samples/min). The ‘Scene 1S4’ autism assessment includes a sequence of static and animated stimuli (illustrated below and detailed in Supplementary Figure S7 and Tables S2–S3) developed by JKC. We confirm the permission from actors to appear in these images or that images were purchased by JKC for this use (including in open-access publication), and that we have the permission of JKC to include the images presented here (and in Supplementary Materials) for illustrative purposes including in open-access publication.

#### Setup and calibration

**Figure 1:**
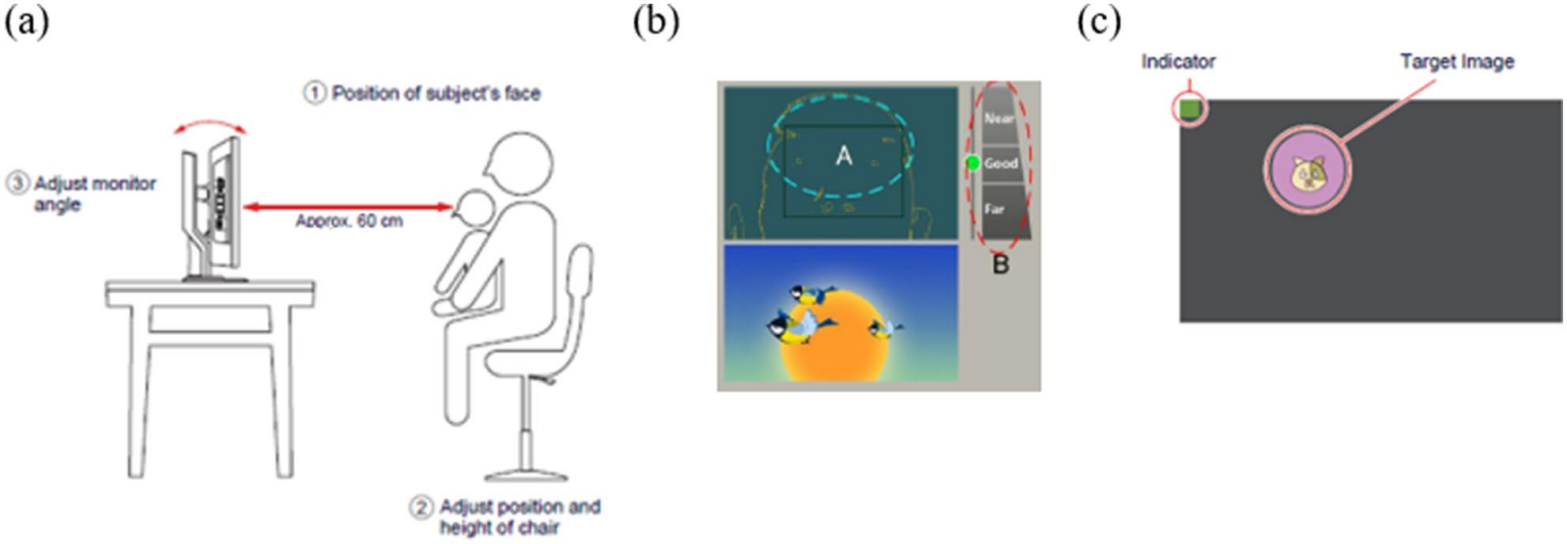
(**a**) Guidance from *Gazefinder* Instruction Manual for setup, (**b**) on-screen visualisation to aide proper positioning of viewer in front of screen, and (**c**) example of attention-grabbing image for calibration check procedure.

While *Gazefinder* eye-tracking was typically conducted towards the end of each appointment after other clinical/behavioural assessments, staff could vary task presentation order to maximise infant engagement across the session. *Gazefinder* assessment was attempted with all consecutively-enrolled infants from the point the apparatus became available at the two study sites*.* Key features of the *Gazefinder* apparatus and recording environment setup are shown in Fig. 1 and Supplementary Figures S3–S6. Infants viewed *Gazefinder* whilst seated on a caregiver’s lap. Caregivers were asked to close their eyes or avert their gaze during the stimulus presentation to avoid inadvertent adult vs. infant gaze data capture. Researchers used the *Gazefinder* on-screen visualisation to check correct infant positioning (also shown in Fig. 1) before triggering a 5-point gaze calibration check (centre- and four peripheral points) ahead of the main ‘Scene 1S4’ stimulus presentation.

*Gazefinder* software was initially configured to begin ‘Scene 1S4’ presentation immediately following the calibration check sequence, without any opportunity for administering personnel to confirm calibration success. An examination of overall data capture (i.e., % tracking rate) for the first 24 infants assessed identified three instances of 0% tracking rate, suggesting undetected unsuccessful initial gaze calibration for these infants. The manufacturer made a minor software adjustment so that ‘Scene 1S4’ would only proceed to the main assessment if *Gazefinder* detected gaze to ≥ 3 (of 5) calibration points, including centre and at least two peripheral points (one top- and one bottom-of-screen). If not successful, *Gazefinder* would automatically repeat the calibration sequence (and only proceed to testing if gaze detected to ≥ 3 calibration points on this second attempt). If calibration was still unsuccessful, the assessment was automatically discontinued alerting the clinician to the issue. The clinician could then use their judgement about whether to reattempt assessment right away—via manual restart, including re-checking infant positioning using the on-screen visualisation, and engaging a new two-phase calibration attempt—or move to other tasks of the broader protocol before attempting *Gazefinder* eye-tracking again later in the session.

No maximum number of calibration attempts was specified, though the main animation sequence was only ever presented once per infant (i.e., if an infant’s engagement seemed to wane following a successful initial calibration and commenced assessment, staff never discontinued the assessment mid-way to begin again later). Supplementary Figures S1–S2 outline the original and modified procedures for establishing gaze calibration before commencing the main assessment.

#### ‘Scene 1S4’ autism assessment sequence

**Figure 2:**
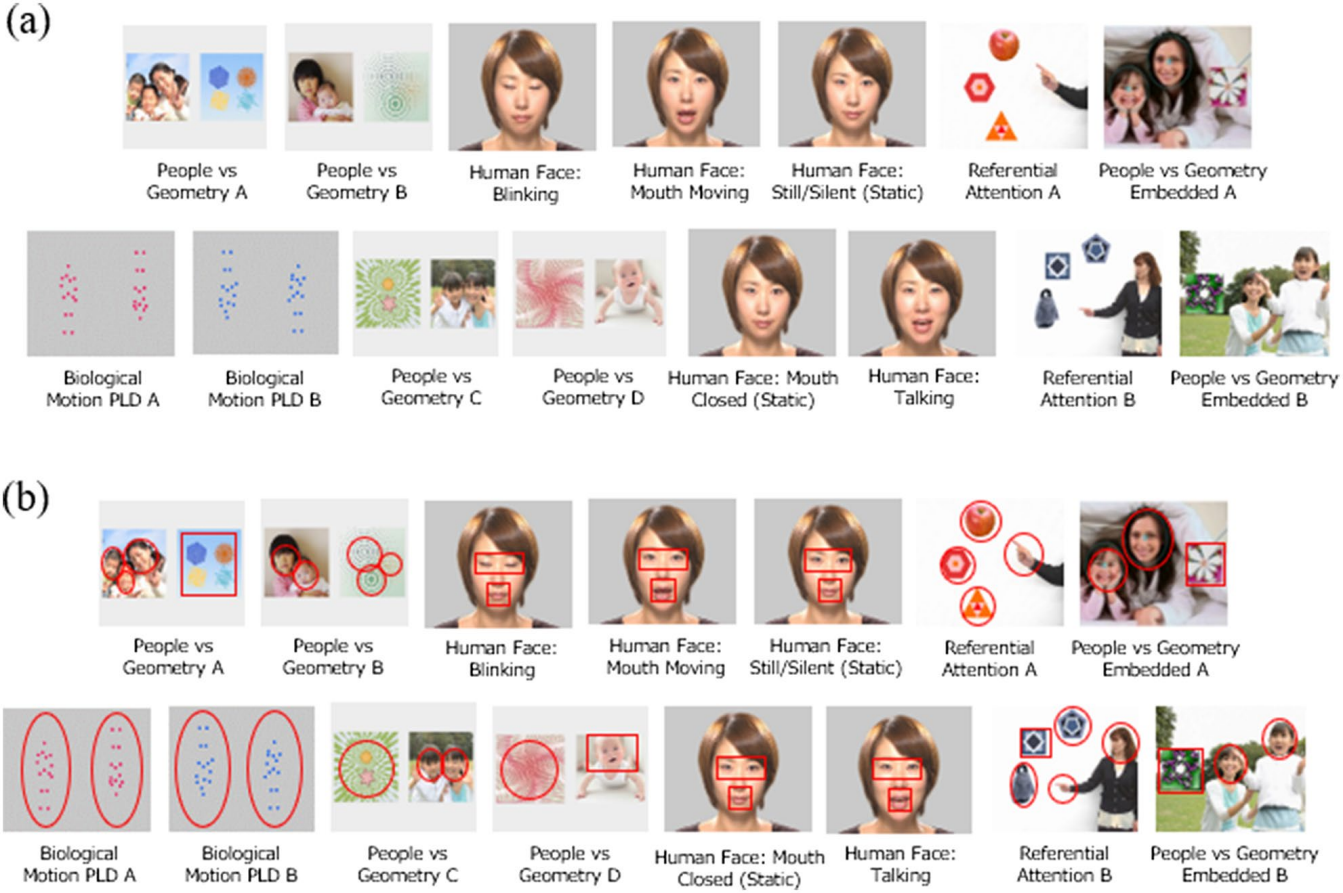
(**a**) Key trials within *Gazefinder* ‘Scene 1S4’ autism assessment sequence, in presentation order (interspersed with attention-grabbers, not shown). (**b**) Pre-specific ROIs within each trial, representing paired social vs. non-social scenes or more vs. less socially-salient elements.

Each infant viewed the identical ‘Scene 1S4’ animation (shown in Fig. 2; detailed in Supplementary Table S3); the same stimulus sequence used in previous studies with older child and adolescent/adult participants^18,36^. This ~ 2-min sequence includes short animations across the following four stimulus types, with *Gazefinder* software automatically summarising individual’s gaze fixations to pre-specified ROIs:

- *Eyes* vs. *mouth* of a human face: one animated trial with eyes blinking (5 s), one animated trial with mouth opening and closing silently (2 s), two static trials (5 and 4 s, respectively), and one animated trial including speech (actress says [spoken in Japanese] “Hello. What is your name? “Let’s play together”; 7 s);
- *People* vs. *geometry*: four trials with same-sized stimuli (range 4.7–5 s per trial; counterbalanced for left vs. right side of screen), and two trials with smaller geometric patterns embedded within larger social scenes (8 s per trial; also counterbalanced for side of screen);
- *Upright* vs. *inverted* biological motion point-light type display (PLD): two trials (counterbalanced for left vs. right side of screen; 5–6 s per trial) with animation accompanied by instrumental song; and
- Referential attention probes: two trials each including a *target*, *two distractor* objects, and a referential *agent*; one trial showed only the pointing hand of the referential agent, while the other showed the full agent torso; 4 s per trial; and accompanied by “What is this?”).

Key trials were preceded by an attention-grabbing central stimulus—a cartoon animal motioning and saying (in Japanese) “Look, look” (1 s per trial; not shown in Fig. 2 and not contributing to gaze data extracted for analysis). Detailed parameters of trial timing and ROIs positioning are provided in Supplementary Tables S2–S3.

### Data analyses

*Gazefinder* software automatically summarised individual infants’ gaze data to the pre-specified ROIs for simple extraction of an encrypted, password-protected .csv file to collate with the other clinical/behavioural. Key *Gazefinder* data of interest here were % Tracking Rate–reflecting the overall amount of any gaze data captured as a proportion of ~ 2-min ‘Scene 1S4’ sequence–within stimulus % tracking rate, as well as gaze fixation durations to pre-specified ROIs within each of the four stimulus types (i.e., averaged across trials where multiple of the same stimulus type).

**Figure 3:**
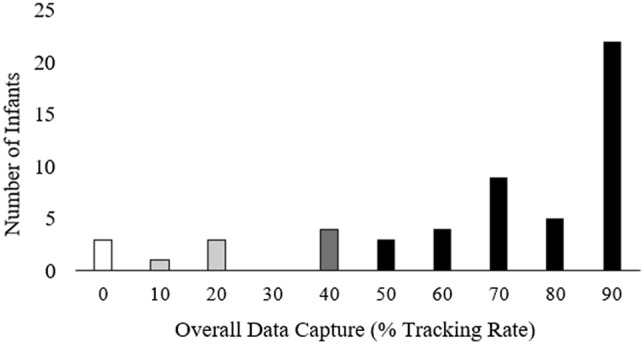
Distribution of overall data capture across ~ 2-min *Gazefinder* “Scene 1S4” stimulus presentation.

Associations between child clinical/behavioural characteristics and the % tracking rate (which was negatively skewed; as shown in Fig. 3) were examined using Spearman correlations. Relative gaze to ROI pairs within each stimulus type (i.e., social vs. non-social ROIs, or more- vs. less socially salient ROIs) was examined using paired-samples *t-*tests. Pearson/Spearman partial correlations—controlling for within-stimulus % tracking rate—were used to explore associations between child clinical/behavioural characteristics and (a) gaze towards each pre-specified ROI, and (b) ratio scores reflecting the *relative* extent of gaze towards social vs. non-social and more vs. less socially-salient ROIs (e.g., eye-vs.-mouth ratio; people-vs.-geometry ratio, etc.). We set no minimum threshold for overall or within-trial % tracking rate, retaining all participants with *any* available data for analyses. We set *α* = 0.05 and applied a false discovery rate (FDR) correction for multiple testing of gaze and clinical assessment association data.

## Results

### Participant characteristics

The sample of infants (74% male) was aged 9–16-months chronologically (and 9–14-months after correction for gestation < 37 weeks [*n* = 4 infants]). Each infant was referred to the larger study by a community health practitioner on the basis of showing three- (26%), four- (26%), or five (48%) of five SACS-R eligibility markers suggesting possible emerging autism^24^. Table 1 presents group-level descriptive clinical/behavioural phenotyping data, and correlations among these measures. Group mean scores suggested moderate-to-high assessed autism behaviours (AOSI total score), broadly age-appropriate non-verbal developmental/cognitive abilities (NVDQ), language abilities delayed by ~ 3 months (receptive and expressive AE scores), and somewhat delayed adaptive skills (VABS ABC). Caregivers of a small subgroup of 10 infants (18.5%) reported that their infant had an older autistic sibling. Post-hoc review of caregiver-reported medical history notes revealed one infant with visual impairment warranting future surgical correction. We nevertheless retained this infant’s data, given *Gazefinder* assessment had been attempted/completed, and returned % Tracking Rate comparable to other infants suggesting no issues with gaze detection or sampling.

**Table 1:** Summary of participant clinical/behavioural characterisation and associations among measures*.*

	N	M (SD)	Range	AOSI	MSEL		VABS	
				Total score	NVDQ	Receptive	Expressive	ABC SS
Chronological Age	54	12.9 (1.83)	9–16	0.02, *p* = 0.888	− 0.29, *p* < 0.05	0.22, *p* = 0.119	0.28, *p* < 0.05	− 0.01, *p* = 0.947
AOSI								
Total score	54	9.50 (4.82)	2–28		− 0.14, *p* = 0.329	− 0.38, *p* = 0.005	− 0.27, *p* < 0.05	− 0.40, *p* < 0.01
MSEL								
NVDQ	53	105.9 (19.7)	61–154			0.43, *p* = 0.002	0.22, *p* = 0.120	0.02, *p* = 0.892
Receptive AE	54	9.9 (3.12)	2–17				0.44, *p* < 0.001	0.27, *p* = 0.064
Expressive AE	54	9.2 (3.02)	4–15					0.40, *p* < 0.01
VABS								
ABC SS	48	83.0 (9.9)	63–103					

*AOSI* Autism Observation Scale for Infants, *MSEL* Mullen Scales of Early Learning, *NVDQ* Non-Verbal Developmental Quotient, *AE* Age Equivalence, *VABS* Vineland Adaptive Behavior Scales, *ABC* Adaptive Behaviour Composite, *SS* Standard Score.

### Eye-tracking feasibility

Figure 3 shows the distribution of overall % tracking rate (range 0–99%) for the 54 infants with whom *Gazefinder* assessment was attempted. As already noted, three of the first 14 assessed returned 0% tracking rate, determined as having been due to examiner error with infant positioning relative to the monitor, resulting in failed calibration that was not detectable prior to proceeding with the main assessment (per original software programming; see Figure S1). Following additional manufacturer guidance on apparatus use and positioning (per Fig. 1 above) and minor software update to abort *Gazefinder* assessment if successful detection of gaze to ≥ 3 calibration points did not occur (see Figure S2), there were no further instances of 0% tracking Rate. Analysable data were therefore obtained for 51 of the 54 infants who completed *Gazefinder* assessment.

Among this final sample of 51 infants with analysable gaze data, overall % tracking rates exceeded 80% for over half of the sample (overall *M* = 77%, *SD* = 22.6%). Four infants returned minimal data (12–27% tracking rates); three infants were noticeably active/unsettled during their assessment (prompting early discontinuation of *Gazefinder* assessment for one due to fussiness, without re-attempt), and the fourth infant appeared attentive suggesting a potential issue with initial calibration/gaze detection. Excluding these four cases of < 30% tracking rate and reanalysing the data yielded substantively similar results (see Supplementary Tables S4–S5). Therefore, we present findings based on the full sample of 51 infants.

Associations between % tracking rate and child age, clinical/behavioural assessment scores, and caregiver-reported temperament measures are shown in Table 2. One single statistically significant association presented with IBQ-R Activity Level, suggesting lower % tracking rate for infants rated by caregivers as being more temperamentally active.

**Table 2:** Association of participant clinical/behavioural characterisation with overall rates of data capture*.*

	N	Data capture
Chronological age	51	*r* = 0.18, *p* = 0.216
AOSI		
Total score	51	*r* = 0.12, *p* = 0.401
MSEL		
Non-verbal DQ	50	*r* = 0.09, *p* = 0.540
Receptive AE	51	*r* = 0.09, *p* = 0.545
Expressive AE	51	*r* = 0.13, *p* = 0.353
VABS		
Adaptive behaviour composite SS	48	*r* = − 0.19, *p* = 0.216
IBQ		
Activity level	49	*r* = − 0.30, *p* = 0.036
Duration of orienting	49	*r* = 0.11, *p* = 0.437
Distress to limitations	49	*r* = 0.02, *p* = 0.869

*AOSI* = Autism Observation Scale for Infants; *MSEL* = Mullen Scales of Early Learning; *NVDQ* = Non-Verbal Developmental Quotient; *AE* = Age Equivalence; *VABS* = Vineland Adaptive Behaviour Scales; *SS* = Standard Score; *IBQ* = Infant Behaviour Questionnaire.

### Preliminary validity

#### Within-trial preferential attention

**Figure 4:**
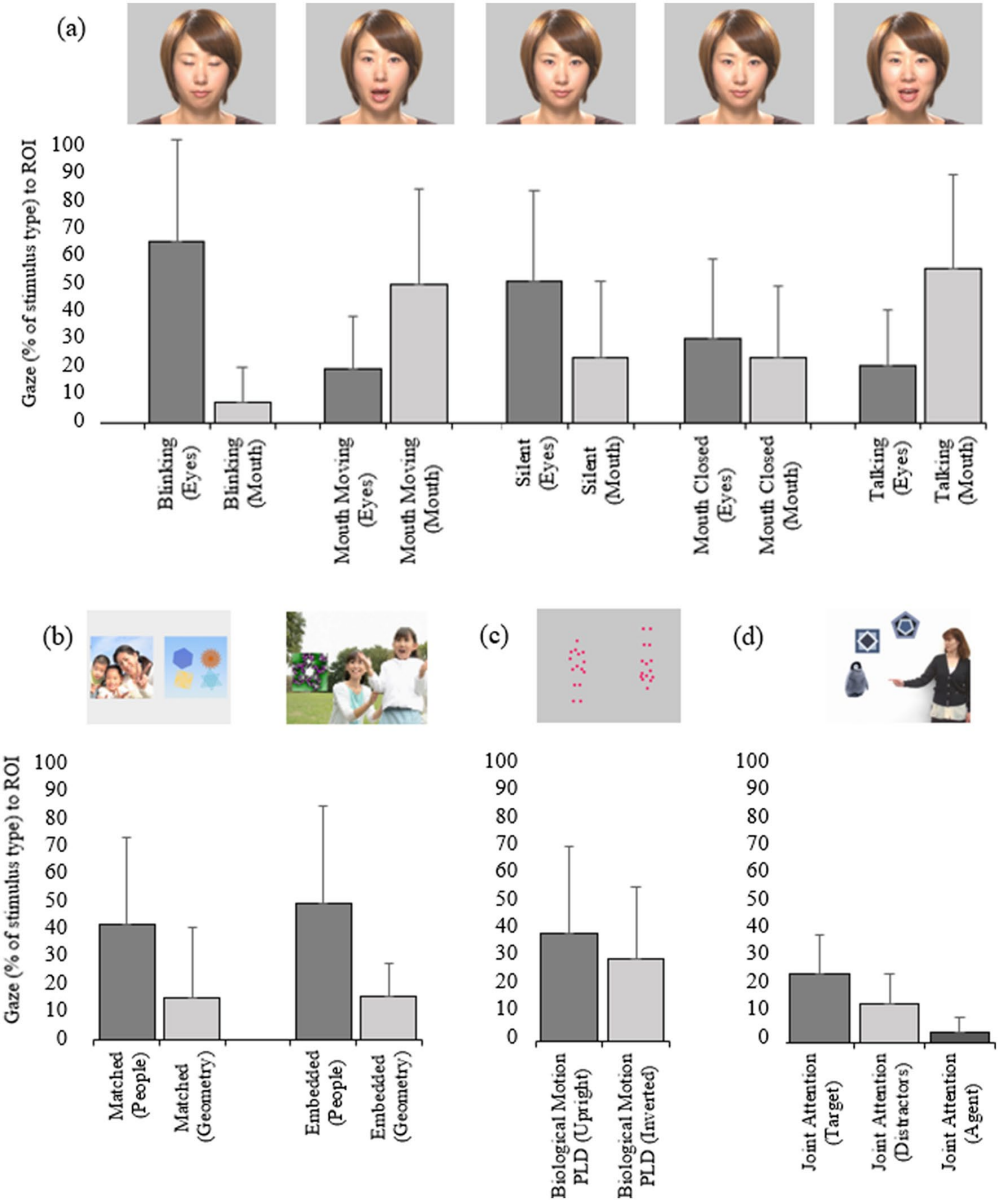
Group mean-level preferential attention (± standard deviation) towards pre-specified social vs. non-social or more vs. less socially-salient ROIs across, four key trial types: (**a**) 5 presentations of human face (varying in static/animated elements); (**b**) 6 scenes pairing people vs. geometry (4 same-size, 2 with geometry embedded within larger social scene); (**c**) 2 biological motion PLD trials; and (**d**) 2 referential attention trials (one showing agent hand only; one showing full torso).

Figure 4 and Table 3 summarise infant gaze data towards pre-specified ROIs within each of five human face trials, and averaged across sets of four same-size people vs. geometry and two geometry-embedded trials, two biological motion PLD trials, and two referential attention probes. As a group, infants showed preferential attention towards eye (vs. mouth) ROIs of the human face during an initial blinking trial and a subsequent static trial, no preferential attention towards either ROI during a second static trial (presented later, separated from the first static trial by other stimulus types; see Fig. 2), and preferential attention towards mouth (vs. eyes) ROIs during two animated trials with mouth moving (silently and whilst talking).

**Table 3:** Summary of gaze towards ROIs across four trial types and between-region significance test*.*

	Group mean level gaze to ROIs		Difference
Human face	Eyes	Mouth	
Trial 1: blinking (silent)	0.60 (0.34) 0.00–1.00	0.07 (0.11) 0.00–0.38	*t*(50) = 10.14, *p* < 0.001, *d* = 1.59
Trial 2: mouth moving (silent)	0.18 (0.18) 0.00–0.82	0.46 (0.32) 0.00–0.89	*t*(50) = 5.79, *p* < 0.001, *d* = 0.82
Trial 3: static	0.47 (0.30) 0.00–1.00	0.22 (0.25) 0.00–0.88	*t*(50) = 3.85, *p* < 0.001, *d* = 0.80
Trial 4: static	0.28 (0.27) 0.00–0.97	0.22 (0.24) 0.00–0.88	*t*(50) = 1.17, *p* = 0.248, *d* = 0.17
Trial 5: talking	0.19 (0.18) 0.00–0.80	0.51 (0.31) 0.00–1.00	*t*(50) = 5.75, *p* < 0.001, *d* = 0.99
People vs. geometry	People	Geometry	
Same-size (4 trials averaged)	0.40 (0.21) 0.00–0.77	0.14 (0.12) 0.00–0.49	*t*(50) = 7.07, *p* < 0.001, *d* = 1.22
Embedded (2 trials averaged)	0.48 (0.29) 0.00–0.94	0.15 (0.18) 0.00–0.75	*t*(50) = 6.07, *p* < 0.001, *d* = 1.00
Biological motion PLD	Upright Figure	Inverted Figure	
(2 trials averaged)	0.38 (0.19) 0.00–0.79	0.29 (0.19) 0.00–0.62	*t*(50) = 2.71, *p* = 0.009, *d* = 0.39
Referential attention	Target	Distractor	
(2 trials)	0.13 (0.10) 0.00–0.38	0.04 (0.05) 0.00–0.22	*t*(50) = 5.99, *p* < 0.001, *d* = 0.82
	Agent face/hand		
	0.23 (0.14) 0.00–0.48	(vs. target)	*t*(50) = 5.01, *p* < 0.001, *d* = 0.68

*ROIs*  regions of interest.

As a group, infants also showed preferential attention towards people vs. geometry ROIs across both same-size and geometry-embedded trial types, and towards upright vs. inverted ROIs within biological motion PLD trials. During referential attention probes, infants showed more gaze towards target vs. distractor object ROIs, but most gaze towards referential agent ROIs (pointing hand, and face when visible).

#### Associations of gaze and emerging autism behaviours

Table 4 shows correlations between infant’s scores on a measure of emerging autism signs (AOSI total scores) and gaze data to the pre-specified social and non-social or more vs. less socially-salient ROIs. AOSI total scores were significantly and moderately strongly correlated with gaze towards non-social (i.e., geometry) ROIs both within same-size (*r* = 0.50) and geometry-embedded trial types (*r* = 0.44). AOSI total scores were also moderately strongly correlated with gaze towards social (i.e., person) ROIs within these same trials, but this was not statistically significantly following FDR correction for multiple comparisons. There was no significant association of AOSI total scores with gaze towards ROIs in human face, biological motion, or referential attention trials.

**Table 4:** Associations between gaze data to ROIs and scores on clinical/behavioural phenotyping measures for 51 infants with any available gaze tracking data*.*

ROI	AOSI		MSEL		VABS		MSEL			
	Total score		NVDQ		ABC SS		Receptive AE		Expressive AE	
Human face										
Blinking										
Eyes	*r* = 0.16	*p* = 0.539	*r* = − 0.06	*p* = 0.964	*r* = 0.26	*p* = 0.410	*r* = − 0.09	*p* = 0.695	*r* = 0.08	*p* = 0.712
Mouth	*r*_*s*_ = − 0.19	*p* = 0.327	*r*_*s*_ = 0.08	*p* = 0.943	*r*_*s*_ = − 0.14	*p* = 0.838	*r*_*s*_ = 0.12	*p* = 0.771	*r*_*s*_ = 0.16	*p* = 0.576
Mouth moving										
Eyes	*r*_*s*_ = 0.10	*p* = 0.653	*r*_*s*_ = − 0.15	*p* = 0.943	*r*_*s*_ = 0.04	*p* = 0.881	*r*_*s*_ = − 0.23	*p* = 0.580	*r*_*s*_ = 0.03	*p* = 0.917
Mouth	*r*_*s*_ = − 0.22	*p* = 0.286	*r*_*s*_ = 0.17	*p* = 0.943	*r*_*s*_ = − 0.17	*p* = 0.838	*r*_*s*_ = 0.08	*p* = 0.771	*r*_*s*_ = − 0.02	*p* = 0.936
Silent/still (static)										
Eyes	*r* = 0.16	*p* = 0.539	*r* = − 0.13	*p* = 0.852	*r* = 0.22	*p* = 0.443	*r* = − 0.21	*p* = 0.300	*r* = − 0.12	*p* = 0.633
Mouth	*r*_*s*_ = − 0.03	*p* = 0.861	*r*_*s*_ = 0.06	*p* = 0.943	*r*_*s*_ = − 0.16	*p* = 0.838	*r*_*s*_ = 0.28	*p* = 0.580	*r*_*s*_ = 0.19	*p* = 0.563
Mouth closed (static)										
Eyes	*r* = 0.04	*p* = 0.768	*r* = 0.11	*p* = 0.852	*r* = 0.20	*p* = 0.443	*r*_*s*_ = 0.01	*p* = 0.955	*r*_*s*_ = 0.20	*p* = 0.633
Mouth	*r*_*s*_ = 0.17	*p* = 0.400	*r*_*s*_ = − 0.14	*p* = 0.943	*r*_*s*_ = − 0.20	*p* = 0.838	*r*_*s*_ = − 0.08	*p* = 0.771	*r*_*s*_ = − 0.08	*p* = 0.749
Talking										
Eyes	*r*_*s*_ = 0.09	*p* = 0.653	*r*_*s*_ = − 0.17	*p* = 0.943	*r*_*s*_ = 0.13	*p* = 0.838	*r*_*s*_ = − 0.12	*p* = 0.771	*r*_*s*_ = − 0.09	*p* = 0.749
Mouth	*r* = − 0.04	*p* = 0.768	*r* = 0.01	*p* = 0.965	*r* = 0.00	*p* = 0.991	*r* = 0.13	*p* = 0.612	*r* = 0.09	*p* = 0.712
People vs geometry										
Same-size										
People	*r* = − 0.37	*p* = 0.070	*r* = − 0.01	*p* = 0.965	*r* = 0.09	*p* = 0.626	*r* = 0.34	*p* = 0.070	*r* = 0.16	*p* = 0.633
Geometry	*r*_*s*_ = **0.50**	*p* < **0.05**	*r*_*s*_ = − 0.03	*p* = 0.943	*r*_*s*_ = − 0.06	*p* = 0.881	*r*_*s*_ = − 0.27	*p* = 0.580	*r*_*s*_ = − 0.13	*p* = 0.668
Embedded										
People	*r*_*s*_ = − 0.35	*p* = 0.056	*r*_*s*_ = − 0.01	*p* = 0.956	*r*_*s*_ = 0.11	*p* = 0.838	*r*_*s*_ = 0.18	*p* = 0.643	*r*_*s*_ = 0.08	*p* = 0.749
Geometry	*r*_*s*_ = **0.44**	*p* = **0**.**05**	*r*_*s*_ = 0.10	*p* = 0.943	*r*_*s*_ = − 0.29	*p* = 0.838	*r*_*s*_ = − 0.12	*p* = 0.771	*r*_*s*_ = − 0.24	*p* = 0.485
Biological motion PLDs										
Upright figure	*r* = 0.13	*p* = 0.539	*r* = − 0.27	*p* = 0.457	*r* = 0.09	*p* = 0.626	*r* = − 0.21	*p* = 0.300	*r* = 0.04	*p* = 0.862
Inverted figure	*r* = − 0.14	*p* = 0.539	*r* = 0.02	*p* = 0.965	*r* = − 0.28	*p* = 0.410	*r* = 0.09	*p* = 0.695	*r* = − 0.15	*p* = 0.633
Referential attention										
Agent (face/hand)	*r* = − 0.07	*p* = 0.754	*r* = − 0.22	*p* = 0.457	*r* = 0.18	*p* = 0.443	*r* = − 0.02	*p* = 0.955	*r* = − 0.13	*p* = 0.633
Target	*r* = − 0.12	*p* = 0.539	*r* = 0.21	*p* = 0.457	*r* = − 0.17	*p* = 0.443	*r* = 0.34	*p* = 0.070	*r* = 0.35	*p* = 0.110
Distractors	*r*_*s*_ = 0.25	*p* = 0.205	*r*_*s*_ = − 0.06	*p* = 0.943	*r*_*s*_ = 0.05	*p* = 0.881	*r*_*s*_ = − 0.02	*p* = 0.915	*r*_*s*_ = − 0.22	*p* = 0.556

Significant values are in [bold].

All correlations are partial (controlling for within-stimulus % tracking rate) and *p* values are FDR corrected. *r* Pearson correlation, *r*_*s*_ Spearman’s correlation, *ROI* region of interest, *AOSI* Autism Observation Scale for Infants, *MSEL* Mullen Scales of Early Learning, *NVDQ* Non-Verbal Developmental Quotient, *VABS* Vineland Adaptive Behavior Scales, *ABC* adaptive behaviour composite, *AE* age equivalence.

Table 5 shows additional correlations between infants’ AOSI total scores and gaze data computed as proportionate measures (i.e., to people vs. geometry ROIs; to eyes vs. mouth ROIs of human face, etc.). Again, AOSI total scores were significantly and moderately strongly correlated with the proportionate index of gaze to people vs. geometry, across both matched size (*r* = − 0.40) and geometry-embedded trials (*r* = − 0.41). This was such that infants showing more signs of autism directed more gaze to concurrently presented geometry *relative* to people. Again, there were no significant correlations between AOSI Total Scores and proportionate gaze measures (to social vs. non-social, or more vs. less socially-salient ROIs) in human face, biological motion, or referential trials.

**Table 5:** Associations between gaze data to paired ROI (ratios) and scores on clinical/behavioural phenotyping measures for 51 infants with any available gaze tracking data*.*

	ROI	AOSI		MSEL		VABS		MSEL			
		Total score		NVDQ		ABC SS		Receptive AE		Expressive AE	
Human face											
Blinking											
Eyes vs mouth		*r*_*s*_ = 0.20	*p* = 0.311	*r*_*s*_ = − 0.11	*p* = 0.943	*r*_*s*_ = 0.14	*p* = 0.838	*r*_*s*_ = − 0.10	*p* = 0.771	*r*_*s*_ = − 0.16	*p* = 0.576
Mouth moving											
Eyes vs mouth		*r*_*s*_ = 0.23	*p* = 0.280	*r*_*s*_ = − 0.15	*p* = 0.943	*r*_*s*_ = 0.08	*p* = 0.881	*r*_*s*_ = − 0.12	*p* = 0.771	*r*_*s*_ = − 0.05	*p* = 0.887
Silent/still (static)											
Eyes vs mouth		*r*_*s*_ = 0.04	*p* = 0.846	*r*_*s*_ = − 0.04	*p* = 0.943	*r*_*s*_ = 0.21	*p* = 0.838	*r*_*s*_ = − 0.25	*p* = 0.580	*r*_*s*_ = − 0.13	*p* = 0.668
Mouth closed (static)											
Eyes vs mouth		*r*_*s*_ = − 0.10	*p* = 0.653	*r*_*s*_ = − 0.05	*p* = 0.943	*r*_*s*_ = 0.13	*p* = 0.838	*r*_*s*_ = 0.02	*p* = 0.915	*r*_*s*_ = 0.18	*p* = 0.563
Talking											
Eyes vs mouth		*r*_*s*_ = 0.05	*p* = 0.846	*r*_*s*_ = − 0.14	*p* = 0.943	*r*_*s*_ = 0.10	*p* = 0.858	*r*_*s*_ = − 0.05	*p* = 0.827	*r*_*s*_ = − 0.01	*p* = 0.971
People vs geometry											
Same-size											
People vs geometry		*r*_*s*_ = − **0.40**	*p* < **0.05**	*r*_*s*_ = − 0.04	*p* = 0.943	*r*_*s*_ = − 0.01	*p* = 0.963	*r*_*s*_ = 0.20	*p* = 0.643	*r*_*s*_ = 0.09	*p* = 0.749
Embedded											
People vs geometry		*r*_*s*_ = − **0.41**	*p* < **0.05**	*r*_*s*_ = − 0.09	*p* = 0.943	*r*_*s*_ = 0.25	*p* = 0.838	*r*_*s*_ = 0.09	*p* = 0.771	*r*_*s*_ = 0.19	*p* = 0.563
Biological motion PLDs											
Upright vs inverted		*r* = 0.17	*p* = 0.539	*r* = − 0.09	*p* = 0.908	*r* = 0.15	*p* = 0.487	*r* = − 0.28	*p* = 0.163	*r* = 0.03	*p* = 0.862
Referential attention											
Target/person vs distractors		*r*_*s*_ = − 0.27	*p* = 0.200	*r*_*s*_ = − 0.01	*p* = 0.956	*r*_*s*_ = 0.09	*p* = 0.858	*r*_*s*_ = 0.06	*p* = 0.785	*r*_*s*_ = 0.25	*p* = 0.485
Target vs person/distractors		*r*_*s*_ = 0.09	*p* = 0.653	*r*_*s*_ = 0.23	*p* = 0.943	*r*_*s*_ = − 0.03	*p* = 0.881	*r*_*s*_ = 0.18	*p* = 0.643	*r*_*s*_ = 0.30	*p* = 0.485
Target vs distractors		*r*_*s*_ = − 0.26	*p* = 0.205	*r*_*s*_ = 0.03	*p* = 0.943	*r*_*s*_ = 0.04	*p* = 0.881	*r*_*s*_ = 0.09	*p* = 0.771	*r*_*s*_ = 0.26	*p* = 0.485

Significant values are in [bold].

All correlations are partial (controlling for within-stimulus % tracking rate) and *p* values are FDR corrected. *r* Pearson correlation, *r*_*s*_ Spearman’s correlation, *ROI* Region of Interest, *AOSI* Autism Observation Scale for Infants, *MSEL* Mullen Scales of Early Learning, *NVDQ* Non-Verbal Developmental Quotient, *VABS* Vineland Adaptive Behavior Scales, *ABC* adaptive behaviour composite, *AE*  age equivalence.

#### Associations of gaze and other clinical phenotyping measures

Also included in Tables 4 and 5 are correlations between measures of infants’ broader developmental/cognitive abilities (MSEL NVDQ and Receptive and Expressive Language AE scores) and adaptive skills (VABS ABC SS) and gaze to pre-specified ROIs and computed proportionate ROI measures. Neither NVDQ nor ABC SS was significantly correlated with gaze data towards any ROI within any stimulus type. There was a suggested moderate-to-strong association between Receptive Language AE and gaze towards the target object ROI during the referential attention probes that was statistically non-significant, however, with correction for multiple comparisons.

## Discussion

Autism diagnosis commonly occurs between age 3 and 5 years^37,38^, but shows stability from 24-months^39^. Neural indicators of emerging autism can be detected in early life^40^ ahead of overt behavioural signs which often appear by the first birthday^21^. A protracted assessment and monitoring process is common across toddlerhood and into early childhood, however, with clinical judgement requiring the confident integration of information across directly observed and informant-reported behaviour, whilst taking into account developmental context and known phenotypic heterogeneity^4^. Eye-tracking technology could expedite clinical decision-making by offering objective indication of autism-associated differences in visual social attention.

Some eye-tracking technologies require substantial user training, involve the time-consuming administration of stimuli across multiple trials, types, and/or paradigms, and can be relatively invasive if involving the use of goggles or head-mounted displays. All of these are important barriers to scalable clinical use. *Gazefinder* is a non-invasive eye-tracking system designed for easy off-the-shelf use by non-technical personnel. Its ‘Scene 1S4’ animation sequence offers a standardised, brief autism assessment protocol capturing gaze to pre-specified ROIs—social vs. non-social scenes, or more vs. less socially-salient within-scene elements—across four key stimulus types: trials showing a static or animated human face, trials tapping preferential attention to people vs. geometry, biological motion PLDs, and referential attention probes. Previous research has shown the potential for *Gazefinder* to support clinical autism decision-making, differentiating autistic from non-autistic individuals in childhood and adolescence/adulthood^17,19,36^. We tested the feasible use of *Gazefinder* with infants referred into a larger research program on the basis of showing signs of emerging autism, integrating the system within an appointment otherwise mirroring an age-appropriate clinical/behavioural autism assessment, conducted by clinical researchers without any particular prior technical eye-tracking expertise. Further, we appraised the preliminary validity of *Gazefinder* data against established and norm-referenced clinical/behavioural characterisation measures, quantifying signs of possible emerging autism, developmental/cognitive and adaptive behaviour skills, in early development.

We hypothesized that infants, as a group, would show broad preferential attention towards social vs. non-social and more vs. less socially-salient within-stimulus ROIs, but that individuals showing more behavioural signs of autism would show relatively greater gaze towards non-social scenes (i.e., geometry [vs. people] in paired preferential attention trials) and less socially-salient elements of scenes (i.e., mouth [vs. eyes] of human face in static and some animated trials; inverted [vs. upright] biological motion in PLD trials; and distractor objects [vs. target object and agent] in referential attention trials). We did not anticipate associations for gaze data with developmental/cognitive ability or adaptive skills measures, but did anticipate that receptive and expressive language skills might associate with gaze towards elements of referential attention trials reflecting shared/joint attention.

### Feasible integration within clinical autism assessment

With minimal training, non-technical clinical researchers feasibly incorporated *Gazefinder* within the broader clinical/behavioural assessment protocol for 54 consecutively-referred infants attending appointments from the time the system became available for use at two study sites. A small number of early failed assessments returning no data despite evident infant attentiveness (i.e., 0% Tracking Rate identified at assessment completion) indicated a potential systematic issue highlighting the importance of proper infant positioning relative to the screen, and benefit of a minor software adjustment that was made, following which there was no further experience of failed assessment.

Overall data capture (i.e., % tracking rate) was generally very high, over 80% for half of the sample; similar to previously-reported *Gazefinder* data capture tested with 4-to 11-month-olds infants sampled from the general population (sample mean 81% tracking rate^19^), and well above rates reported elsewhere in studies with infants using other eye-tracking systems^41^. Only four of the 51 successfully assessed infants in the current study returned < 30% overall tracking rates, with staff notes suggesting these could mostly be explained by waning inattention/increased distractibility during the stimulus presentation. Indeed, across the sample, among various child characteristics examined, only parent-reported temperamental activity level showed any association with % tracking rate. We therefore conclude that *Gazefinder* eye-tracking was generally feasibly implemented by clinical research staff and well-tolerated by infants with identified signs of possible emerging autism, referred around the first birthday.

### Some preliminary validity evidence for infant assessment

Evidence of group mean-level preferential attention was as expected. There were strong preferential effects for social vs. non-social scenes (i.e., people vs. geometry in paired scenes, whether matched size or geometry embedded within larger social scenes) and for more vs. less socially-salient within-scene elements within human face trials (i.e., eyes vs. mouth in an initial static trial and an animated blinking trial; but towards mouth vs. eyes in animated trials with mouth moving silently and/or when talking) and referential attention trials (i.e., most attention directed towards referential agent, followed by more attention towards target vs. distractor objects). There was also significantly greater gaze, at infant group mean-level gaze, towards upright vs. inverted biological motion PLDs animations, with small-to-moderate effect size. These data further evidence for the validity of the key stimulus types included in the ‘Scene 1S4’ *Gazefinder* autism assessment animation, consistent with previous accounts from use of this system with other samples/populations^17–19^, and from these paradigms presented to infants using other eye-tracking technologies^10,11^.

The current data offered more limited evidence of concurrent validity in the observed associations between infant gaze data and AOSI scores as an established measure of early behavioural autism signs. Infants expressing more autism behaviours directed more gaze to geometry ROIs in paired scenes, including both when computed as raw gaze towards the non-social element and a proportionate metric of gaze relative to the people ROIs. There was no association of autism behaviours, however, with gaze specifically towards social ROIs (i.e., people) within these same stimuli and, furthermore, no other association of assessed autism behaviours with gaze during human face, biological motion PLD, or referential attention trials.

The association of emerging autism behaviours with greater gaze towards geometry (alone, and vs. people) is consistent with a behavioural profile of social attention differences and circumscribed interests considered to be characteristic of autism^1^ and the prior evidence of visual social attention differences in autism established on the basis of eye-tracking research using *Gazefinder*^18^ and other technologies^14^. Indeed, heightened gaze to non-social ROIs has been shown to robustly discriminate autistic from non-autistic children and adults^10,11^, and a visual preference for geometric patterns in infancy identified as a promising biomarker for later autism outcome^14^.

The lack of observed association of autism behaviours and gaze towards eye vs. mouth ROIs within presentations of a human face is somewhat surprising given reduced eye contact is a cardinal feature of autism, and prior evidence of differential attention to facial features in autism^10,11^. However, research suggests that infants later diagnosed with autism initially show eye fixation patterns similar to typical levels, but this gradually declines by the age of 24 months, when reductions compared to children without autism become maximally apparent^42^. Similarly, the lack of association of autism behaviours with gaze towards the referential agent and target object (vs. distractors) in referential attention probes is surprising in light of the child and adolescent eye-tracking literature^43–45^. However, evidence of gaze-following differences in infants at higher likelihood or in infants who later received an autism diagnosis has not always been found^46–48^. Consequently, the *Gazefinder* human face and referential attention stimuli might prove more effective in identifying visual attention differences related to autism at later developmental stages^17,18^.

Moreover, while other behavioural studies suggest autistic people autism may be less sensitive to the social information conveyed in human movement (e.g., emotions, intentions)^49^, we found no evidence associating emerging autism behaviours with gaze to biological motion PLDs in this infant sample. This null finding is consistent with Fujioka et al.’s previous report following *Gazefinder* eye-tracking assessment with autistic and non-autistic adolescents/adults^18^ but contrasts with other studies finding reduced fixation towards similar biological motion PLD stimuli in autistic children aged 2–7 years^50–52^. Limited utility of the specific biological motion PLD trials within the *Gazefinder* ‘Scene 1S4’ protocol may therefore be suggested for autism-related visual social attention assessment.

Finally, we observed virtually no association between gaze data and measures of cognitive/developmental functioning, suggesting the potential specificity of the *Gazefinder* people vs. geometry stimulus for the assessment of autism-related differences in visual attention. The moderate (albeit non-significant) association of assessed receptive language with gaze towards a referential target aligns with the importance of shared and referential (i.e., dyadic and triadic) attention in language development^20^. The stronger correlation observed here, compared to the link between gaze at the same target and autism-related behaviours, indicates that this stimulus could be more useful in evaluating language abilities rather than autism behaviours, particularly during this early developmental stage.

### Limitations and future directions

This study adds to the growing body of evidence supporting the potential utility of eye-tracking technology in clinical autism assessment protocols, providing proof-of-concept for *Gazefinder* as a standardized, brief, user friendly—and highly scalable—option. Limitations of our study include the cross-sectional design and relatively small sample of participating infants. We have included repeated *Gazefinder* assessments within our prospective follow-up of this clinically-indicated cohort into early childhood, which will permit further evaluation of: (a) convergent validity against concurrent clinical/behavioural measures taken at later child ages (with larger participant samples, given the system was only available mid-way through the study recruitment phase and first assessment, reported here), and (b) predictive validity from earlier *Gazefinder* assessment to later clinical characterisation and diagnostic outcome.

Another limitation of note is that our implementation of *Gazefinder* was not within a true community clinical autism assessment service; rather, in university/research settings that mirrored a quality community practice. Further, we made minor but non-trivial adjustments to *Gazefinder* software and staff training during the course of the study (see Figures S1–S2). We have transparently reported on our experience of implementing *Gazefinder* within the broader protocol of clinical/behavioural assessments for our research program, and sought to closely mirror potential future clinical use of this system. Specifically, we deployed the *Gazefinder* GP-100EA apparatus off-the-shelf, implemented the standardised ~ 2-min ‘Scene 1S4’ autism assessment without modification, and attempted assessment with all consecutively enrolled infants from the point the system was available at both sites (i.e., regardless of medical history or clinical/behavioural presentation). Moreover, we analysed recorded gaze data to pre-specified ROIs, exported these data directly from the *Gazefinder* units without any post-processing, and retained all participants with any usable data (i.e., regardless of overall % tracking rate).

Finally, while the standardised stimulus presentation is a strength of the *Gazefinder* system, this also introduces the potential for undetectable presentation order effects which might plausibly explain some of our observed null findings. In particular, the diverging pattern of group mean-level preferential attention to eyes vs mouth of a static human face across initial and later presentations of this identical stimulus, and the lack of expected association of assessed autism behaviours and gaze towards less socially-salient ROIs across some trial types. As evident in the detailed ‘Scene 1S4’ stimulus parameters included here and in Supplementary Materials, some between-trial counterbalancing exists for left- vs. right-hand side of screen, even within the brief assessment protocol. Plausibly the costs of inadvertent presentation/order effects may be offset by the comparability benefits of employing a standardised animation across various study samples and populations.

The current data offer some support for the potential of *Gazefinder* with standardised ‘Scene 1S4’ stimulus sequence to offer a scalable objective assessment to support early autism detection. Employing sophisticated data-driven techniques, such as machine learning, could bring significant further insights from this technology and the data it derives automatically from infant and other viewers. Drawing on data from the most informative ROIs and gaze metrics—potentially including fixation durations but also saccade patterns, pupillary responses, etc.^53,54^—such approach could generate an autism prediction algorithm capable of assigning a probability or classification likelihood (e.g., high, moderate, low). Indeed, there has been a gradual increase in research adopting machine learning for eye-tracking data in attempt to classify autistic vs. non-autistic development, with a recent meta-analysis of 24 studies reporting pooled 81% classification accuracy^55^, underscoring the potential from integrating multiple eye-tracking indicators for autism diagnosis. The varied stimulus array and dataset offered by *Gazefinder* offers fertile ground for applying machine learning techniques and the current study represents an initial phase of this pursuit, laying the groundwork for further exploration and the potential scalable implementation of objective early autism identification.

### Supplementary Information

Supplementary Information.

## Data Availability

The datasets generated during and/or analysed during the current study are available from the corresponding author on reasonable request.
