# Supplementary material for: Feasibility of a 2-minute eye-tracking protocol to support the early identification of autism
Source: Sci Rep. 2024 Mar 1;14:5117. doi: 10.1038/s41598-024-55643-z (PMC10907367; doi:10.1038/s41598-024-55643-z)
Supplement: Supplementary file 1 — Supplementary Information. [file 41598_2024_55643_MOESM1_ESM.docx]

**Supplementary Material to**

Feasibility of a Two-Minute Eye-Tracking Protocol to Support the Early Identification of Autism

**Lacey Chetcuti^1^, Kandice J. Varcin^2,3^, Maryam Boutrus^3^, Jodie Smith^1,4^,**

**Catherine A. Bent^1^, Andrew J.O. Whitehouse^3^, Kristelle Hudry^1^***

^1^Department of Psychology, Counselling and Therapy, School of Psychology and Public Health, La Trobe University, Bundoora, Victoria 3086 Australia.

^2^Telethon Kids Institute, The University of Western Australia, Nedlands, Western Australia 6009 Australia.

^3^School of Psychological Science, University of Western Australia, Crawley, Western Australia 6009, Australia.

^4^Macquarie School of Education, Macquarie University, North Ryde, New South Wales 2109, Australia.

^5^School of Allied Health, Human Services and Sport, La Trobe University, Bundoora, Victoria 3086 Australia.

^6^St Giles Society Limited, Burnie, Tasmania 7320 Australia.

^7^JVCKENWOOD Corporation, 3-12 Moriya-cho, Kanagawa-ku, Yokohama-shi, Kanagawa, 221-0022, Japan.

*Corresponding author: A/Prof. Kristelle Hudry, Department of Psychology, Counselling and Therapy, School of Psychology and Public Health, La Trobe University, Bundoora, Victoria 3086 Australia, Email: k.hudry@latrobe.edu.au, Phone: +61 3 9479 5649.

**Supplementary Material Contents**

| **Section** | **Page** |
| --- | --- |
| Figure S1. Procedural tree illustrating the original *Gazefinder* calibration procedure (first *n*=24) | S2 |
| Figure S2. Procedural tree illustrating the revised *Gazefinder* calibration procedure (subsequent n=30) | S3 |
| Table S1. Specifications following Holmsqvist et al.’s (2023) reporting guidelines. | S4 |
| **Details of *Gazefinder* Apparatus and Assessment** |  |
| Figure S3. Specifications from product *Gazefinder* *Instruction Manual^1^* (p. 59 & 62). | S5 |
| Figure S4. Description of gaze detection technology from *Gazefinder* *Instruction Manual^1^* (p. 18) and product *Instruction Manual: Operation^2^* (p. 51-52). | S6 |
| Figure S5. Device setup and viewer positioning guidance from product *Instruction Manual: Operation* (p. 10-11) | S6 |
| Figure S6. Guidance for checking positioning and calibration from *Gazefinder* *Instruction Manual^1^* (p. 27-30) and product *Instruction Manual: Operation^2^* (p. 19-20). | S7 |
| Figure S7. Key stimuli within ‘Scene 1S4’ showing all pre-specified Regions of Interest (ROIs) | S8 |
| Table S2. Detailed specifications of ROIs for initial calibration check | S8 |
| Table S3. Detailed specifications of pre-specified ‘Scene 1S4’ ROIs | S9 |
| Table S4. Associations between gaze data to pre-specified ROIs and scores on clinical/behavioural phenotyping measures for 47 infants with >30% Tracking Rate | S11 |
| Table S5. Associations between gaze data to proportionate gaze data to social vs. non-social ROIs or more vs. less socially-salient ROIs and scores on clinical/behavioural phenotyping measures for 47 infants with >30% Tracking Rate | S12 |


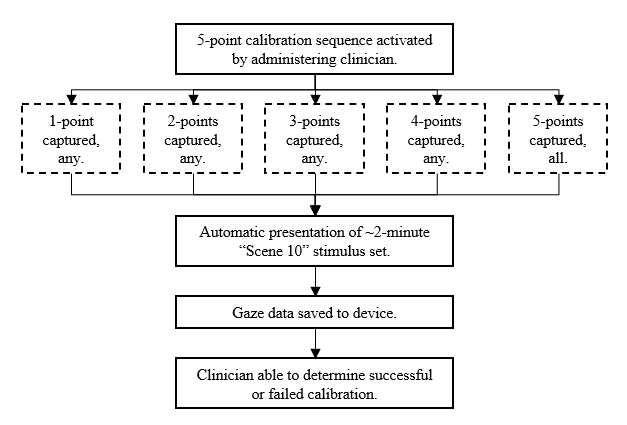


Figure S1. Procedural tree illustrating the original *Gazefinder* calibration procedure (first *n* = 24).


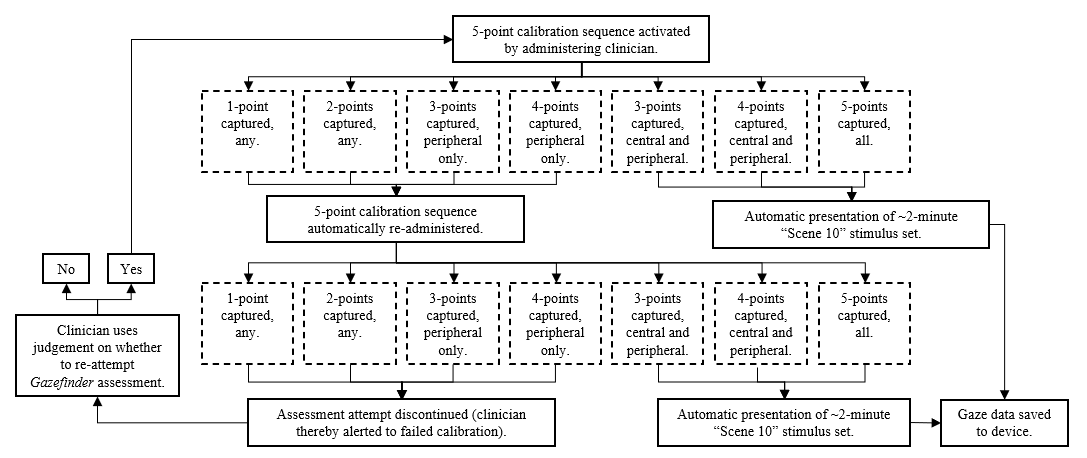


Figure S2. Procedural tree illustrating the revised *Gazefinder* calibration procedure (subsequent *n* = 30).

Table S1. Specifications following Holmsqvist et al.’s (2023) reporting guidelines.

| Reporting aspect | Manuscript Main Text | Supplementary Materials |
| --- | --- | --- |
| 1. Details about the eye-tracker | pg. 3-4 | Figures S3, S4 |
| 1. Sampling frequency | pg. 3 | Figure S3 |
| 1. Description of the setup and geometry | pg. 4 | Figures S3-S5 |
| 1. Description of the recording environment | pg. 4 | Figure S5 |
| 1. The instruction given to participants | pg. 4 | - |
| 1. Empirically determined data quality for the analysed eye-tracker signals | pg. 5 | Figure S6  Table S2 |
| 1. Description of the data processing and analysis steps | pg. 4-5 | - |
| 1. Firmware and software versions | pg. 3-4 | Figures S3, S7 |
| 1. Exclusion criteria, pre- and post-recording | pg. 3, 5 | - |

# Details of *Gazefinder* Apparatus and Assessment

Figures S3 through S6 show details of the *Gazefinder* apparatus (Model GP-100 EA) and guidelines for device setup and participant/viewer positioning, calibration etc. These are taken from the *Gazefinder* *Instruction Manual: Gaze Tracking and Diagnostic System Model GP-100EA^1^* and product *Instruction Manual: Operation^2^.*

**
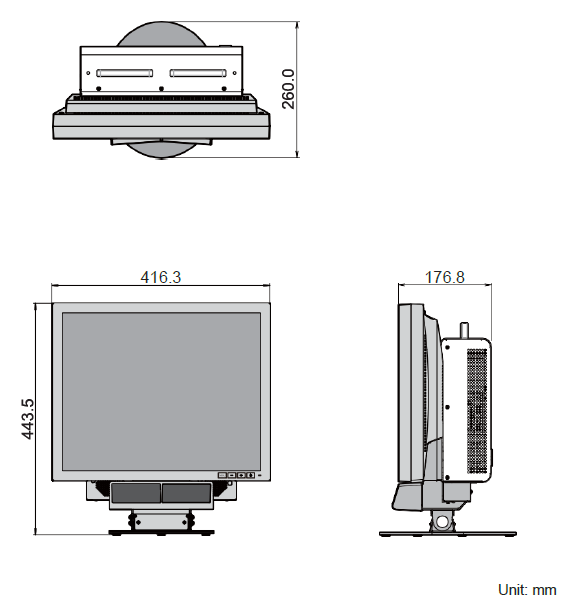
**
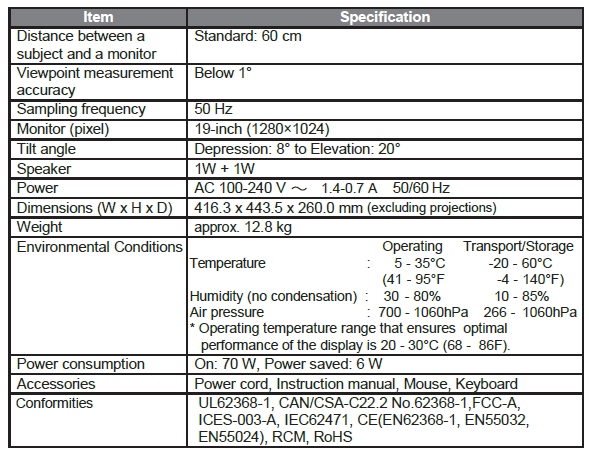
**Gazefinder apparatus description and specifications**

Figure S3. Specifications from *Gazefinder* *Instruction Manual: Gaze Tracking and Diagnostic System Model GP-100EA^1^* (p. 59 & 62).

**Determination of gaze fixations and movement saccades**


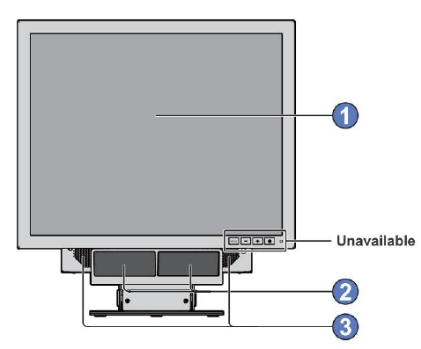


Screen

Stereo Camera/Infrared LED

Speaker


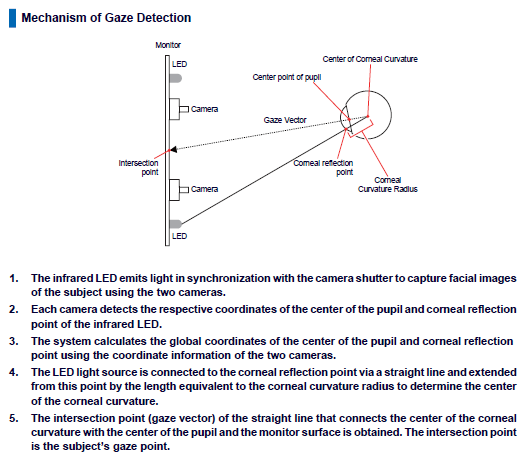

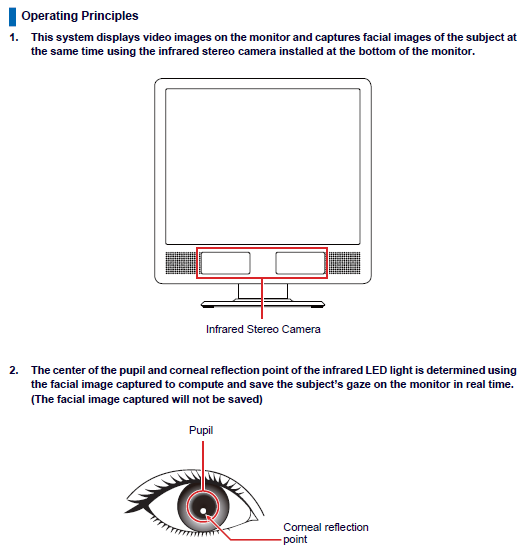


Figure S4. Description of gaze detection technology from *Gazefinder* *Instruction Manual: Gaze Tracking and Diagnostic System Model GP-100EA^1^* (p. 18) and product *Instruction Manual: Operation^2^* (p. 51-52).

**
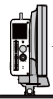
Set-up, positioning check, and five-point calibration procedure**


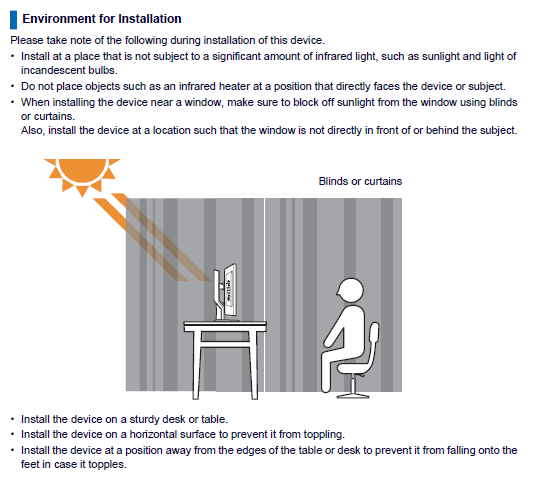

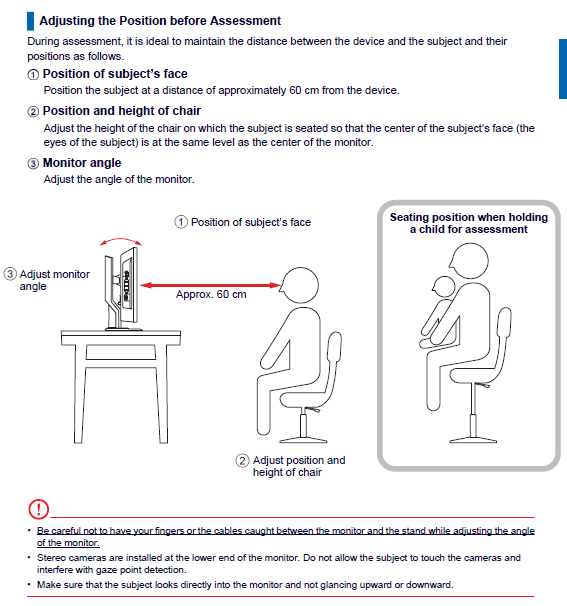


Figure S5. Device setup and viewer positioning guidance from product *Instruction Manual: Operation^2^* (p. 10-11).


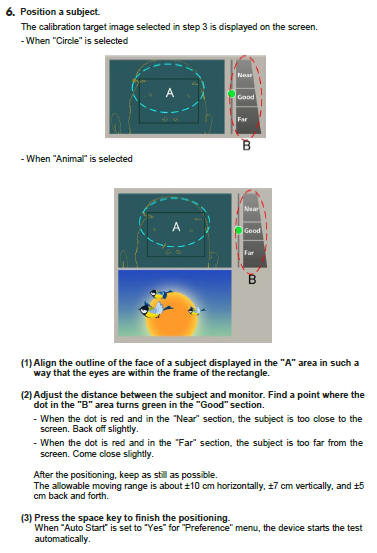

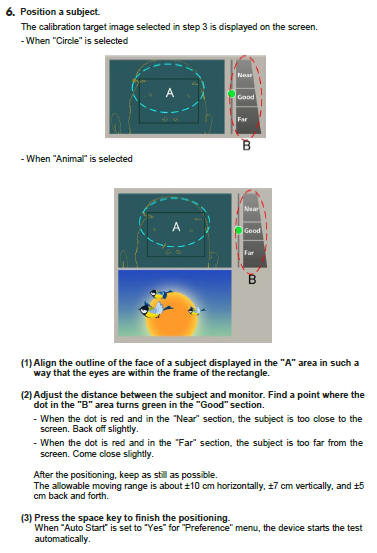

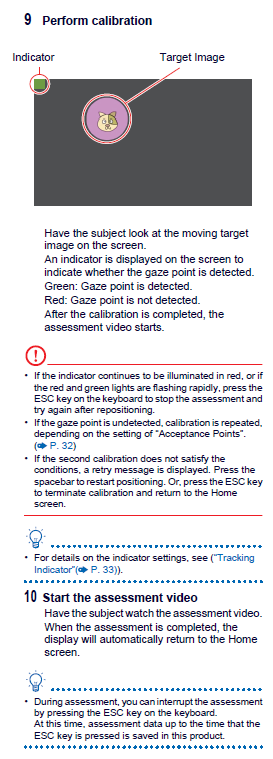

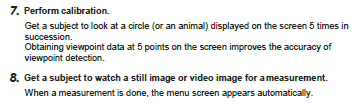

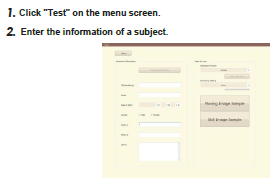

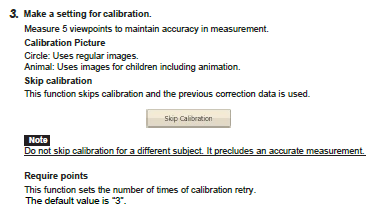

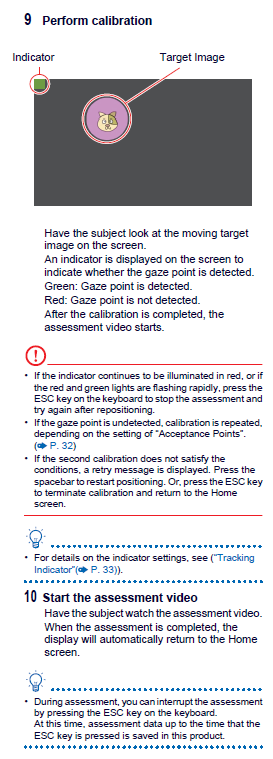

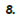


Figure S6. Guidance for checking proper positioning and calibration from *Gazefinder* *Instruction Manual: Gaze Tracking and Diagnostic System Model GP-100EA^1^* (p. 27-30) and product *Instruction Manual: Operation^2^* (p. 19-20).

Sources

1. JVCKENWOOD Corporation (2019). *Gazefinder* *Instruction Manual: Gaze Tracking and Diagnostic System Model GP-100EA*. Kanagawa, Japan. Author
2. JVCKENWOOD Corporation (2022). *Gazefinder* *Instruction Manual: Operation* (GP-200). Kanagawa, Japan. Author


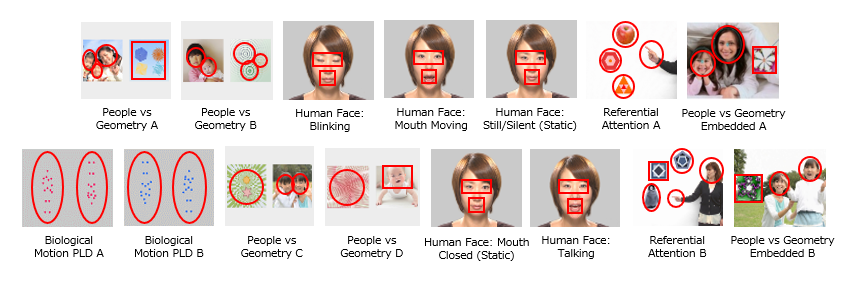


Figure S7. Key stimuli within ‘Scene 1S4’ showing all pre-specified Regions of Interest (ROIs). We confirm the permission from actors to appear in these images or that images were purchased by JKC for this use (including in open-access publication), and that we have the permission of JKC to include the images presented here (and in Supplementary Materials) for illustrative purposes including in open-access publication.

Table S2. Detailed specifications of ROIs for initial calibration check

| **Order** | **Onset Time (ms)** | **ROI Duration (ms)** | **ROI Shape** | **ROI Parameters (pixels)** | | | | **Thumbnail Image** | **ROI Content Summary** |
| --- | --- | --- | --- | --- | --- | --- | --- | --- | --- |
|  |  |  |  | **x-axis** | **y-axis** | **width** | **height** |  |  |
| 1 | 0 | 2,000 | Five Squares | 580 | 454 | 115 | 115 | 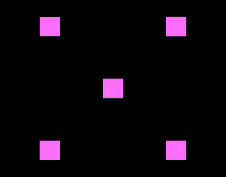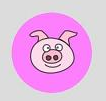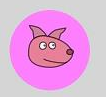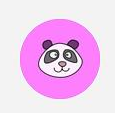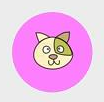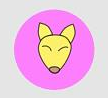 | Centre |
| 2 | 3,000 | 2,000 |  | 225 | 98 | 115 | 115 |  | Top Left |
| 3 | 6,000 | 2,000 |  | 225 | 813 | 115 | 115 |  | Bottom Left |
| 4 | 9,000 | 2,000 |  | 940 | 813 | 115 | 115 |  | Bottom Right |
| 5 | 12,000 | 3,000 |  | 940 | 98 | 115 | 115 |  | Top Right |

Table S3. Detailed specifications of pre-specified ‘Scene 1S4’ ROIs

| **ROI #** | **Trial/Segment Label** | **Onset Time (ms)** | **Stimulus/ROI Duration (ms)** | **ROI Shape** | **ROI Parameters (pixels)** | | | | **Thumbnail Images** | **ROI Content Summary** |
| --- | --- | --- | --- | --- | --- | --- | --- | --- | --- | --- |
|  |  |  |  |  | **x-axis** | **y-axis** | **width** | **height** |  |  |
| 1 | People vs. Geometry (Same Size) A | 5,000 | 5,000 | Three Circles | 28 | 384 | 188 | 247 | 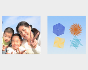 | People |
| 2 |  |  |  |  | 136 | 478 | 199 | 256 |  |  |
| 3 |  |  |  |  | 213 | 278 | 308 | 333 |  |  |
| 4 |  |  |  | Rectangle | 738 | 301 | 493 | 450 |  | Geometry |
| 5 | People vs. Geometry (Same Size) B | 10,300 | 4,700 | Two Circles | 118 | 365 | 229 | 248 | 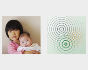 | People |
| 6 |  |  |  |  | 288 | 465 | 220 | 237 |  |  |
| 7 |  |  |  | Three Circles | 757 | 280 | 280 | 280 |  | Geometry |
| 8 |  |  |  |  | 832 | 517 | 260 | 260 |  |  |
| 9 |  |  |  |  | 991 | 433 | 220 | 220 |  |  |
| 10 | Blinking | 17,000 | 5,000 | Two Rectangles | 426 | 397 | 420 | 210 | 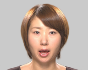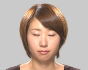 | Eyes |
| 11 |  |  |  |  | 527 | 652 | 210 | 210 |  | Mouth |
| 12 | Mouth Moving | 22,000 | 2,000 | Two Rectangles | 435 | 373 | 426 | 213 | 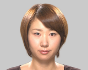 | Eyes |
| 13 |  |  |  |  | 535 | 662 | 213 | 213 |  | Mouth |
| 14 | Static/Silent | 27,000 | 5,000 | Two Rectangles | 431 | 373 | 426 | 213 |  | Eyes |
| 15 |  |  |  |  | 532 | 647 | 213 | 213 |  | Mouth |
| 16 | Referential Attention A | 34,000 | 4,000 | Square | 1080 | 140 | 200 | 200 | 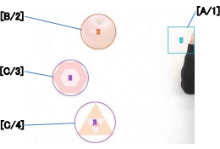 | Pointing Finger |
| 17 |  |  |  | Circle | 419 | 42 | 268 | 268 |  | Target Object |
| 18 |  |  |  | Two Circles | 204 | 388 | 274 | 274 |  | Two Distractors |
| 19 |  |  |  |  | 373 | 707 | 310 | 310 |  |  |
| 20 |  |  |  | Two Circles | 760 | 270 | 320 | 320 | 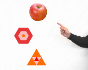 | Pointing Finger |
| 21 |  |  |  |  | 419 | 42 | 268 | 268 |  | Target Object |
| 22 |  |  |  | Two Circles | 204 | 388 | 274 | 274 |  | Two Distractors |
| 23 |  |  |  |  | 373 | 707 | 310 | 310 |  |  |
| 24 | People vs. Geometry (Embedded) A | 40,000 | 8,000 | Square | 910 | 350 | 320 | 320 | 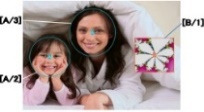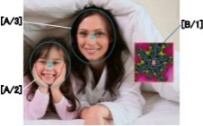 | Geometry |
| 25 |  |  |  | Two Circles | 55 | 341 | 329 | 373 |  | People |
| 26 |  |  |  |  | 379 | 75 | 375 | 447 |  |  |
| 27 |  |  |  | Square | 910 | 350 | 320 | 320 |  | Geometry |
| 28 |  |  |  | Two Circles | 55 | 341 | 329 | 373 |  | People |
| 29 |  |  |  |  | 379 | 75 | 375 | 447 |  |  |
| (continues…) | |  |  |  |  |  |  |  |  |  |
| (continuation) | |  |  |  |  | | | |  |  |
| **ROI #** | **Trial/Segment Label** | **Onset Time (ms)** | **Stimulus/ROI Duration (ms)** | **ROI Shape** | **ROI Parameters (pixels)** | | | | **Thumbnail Images** | **ROI Content Summary** |
|  |  |  |  |  | **x-axis** | **y-axis** | **width** | **height** |  |  |
| 30 | Biological Motion A | 52,000 | 5,000 | Two Circles | 145 | 120 | 420 | 780 | 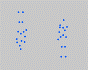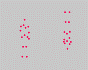 | Upright Figure |
| 31 |  |  |  |  | 770 | 120 | 420 | 780 |  | Inverted Figure |
| 32 | Biological Motion B | 57,000 | 6,000 | Two Circles | 100 | 90 | 420 | 780 |  | Inverted Figure |
| 33 |  |  |  |  | 715 | 130 | 420 | 780 |  | Upright Figure |
| 34 | People vs. Geometry (Same Size) C | 65,000 | 5,000 | Three Circles | 74 | 282 | 470 | 470 | 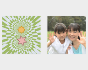 | Geometry Panel |
| 35 |  |  |  |  | 761 | 337 | 241 | 265 |  | People Panel |
| 36 |  |  |  |  | 963 | 354 | 232 | 272 |  |  |
| 37 | People vs. Geometry (Same Size) D | 70,300 | 4,700 | Circle | 72 | 275 | 470 | 470 | 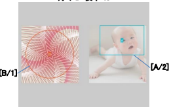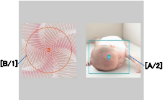 | Geometry Panel |
| 38 |  |  |  | Rectangle | 719 | 408 | 415 | 344 |  | People Panel |
| 39 |  |  |  | Circle | 72 | 275 | 470 | 470 |  | Geometry Panel |
| 40 |  |  |  | Rectangle | 799 | 231 | 431 | 288 |  | People Panel |
| 41 | Mouth Closed (Static) | 80,000 | 4,000 | Rectangle | 444 | 397 | 420 | 210 | 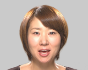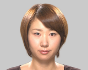 | Eyes Region |
| 42 |  |  |  | Square | 541 | 651 | 210 | 210 |  | Mouth Region |
| 43 | Talking | 84,000 | 7,000 | Rectangle | 425 | 382 | 432 | 216 |  | Eyes Region |
| 44 |  |  |  | Square | 533 | 661 | 216 | 216 |  | Mouth Region |
| 45 | Referential Attention B | 93,000 | 4,000 | Circles | 760 | 300 | 210 | 210 | 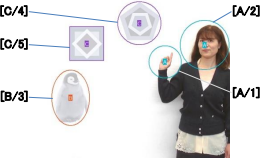 | Referential Agent (Hand & Face) |
| 46 |  |  |  |  | 961 | 137 | 316 | 319 |  |  |
| 47 |  |  |  | Oval | 139 | 456 | 243 | 361 |  | Target Object |
| 48 |  |  |  | Circle & Square | 561 | 17 | 280 | 280 |  | Two Distractors |
| 49 |  |  |  |  | 255 | 179 | 219 | 219 |  |  |
| 50 |  |  |  | Circles | 520 | 560 | 210 | 210 | 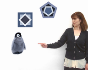 | Referential Agent (Hand & Face) |
| 51 |  |  |  |  | 961 | 137 | 316 | 319 |  |  |
| 52 |  |  |  | Oval | 139 | 456 | 243 | 361 |  | Target Object |
| 53 |  |  |  | Circle & Square | 561 | 17 | 280 | 280 |  | Two Distractors |
| 54 |  |  |  |  | 255 | 179 | 219 | 219 |  |  |
| 55 | People vs. Geometry (Embedded) B | 99,000 | 8,000 | Square | 50 | 350 | 320 | 320 | 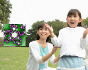 | Geometry Panel |
| 56 |  |  |  | Two Circles | 501 | 320 | 261 | 282 |  | Adult’s Face |
| 57 |  |  |  |  | 874 | 103 | 278 | 297 |  | Child’s Face |

Note. For ROI Parameters, origin of x- and y- axes is top-left corner of screen, and width and height parameters for circles/ovals representing maximal points. We confirm the permission from actors to appear in these images or that images were purchased by JKC for this use (including in open-access publication), and that we have the permission of JKC to include the images presented here (and in Supplementary Materials) for illustrative purposes including in open-access publication.

Table S4. Associations between gaze data to pre-specified ROIs and scores on clinical/behavioural phenotyping measures for 47 infants with >30% Tracking Rate.

|  |  | **AOSI** | | **MSEL** | | **VABS** | | | **MSEL** | | | | | | | |
| --- | --- | --- | --- | --- | --- | --- | --- | --- | --- | --- | --- | --- | --- | --- | --- | --- |
| **Scene/Trial Type** | **ROI** | **Total *^[S]^*** | *q* | **NVDQ** | *q* | **ABC SS** | *q* | | **Receptive AE** | | *q* | | **Expressive AE** | | *q* | |
| **Human Face** |  |  |  |  |  |  |  | |  | |  | |  | |  | |
| Blinking | Eyes | .27 | .226 | -.03 | .967 | .33 | .450 | | -.12 | | .601 | | .07 | | .678 | |
|  | Mouth ***^[S]^*** | -.21 | .371 | .07 | .895 | -.13 | .813 | | .14 | | .551 | | .15 | | .609 | |
| Mouth Moving | Eyes ***^[S]^*** | .20 | .422 | -.09 | .895 | .10 | .813 | | -.30 | | .354 | | .04 | | .840 | |
|  | Mouth | -.24 | .298 | -.03 | .967 | .01 | .944 | | .13 | | .601 | | .13 | | .590 | |
| Silent/Still (Static) | Eyes | .09 | .746 | -.13 | .967 | .23 | .459 | | -.22 | | .403 | | -.13 | | .590 | |
|  | Mouth ***^[S]^*** | -.03 | .957 | .06 | .895 | -.16 | .813 | | .28 | | .354 | | .19 | | .518 | |
| Mouth Closed (Static) | Eyes | .12 | .674 | .14 | .967 | .28 | .459 | | .05 | | .790 | | .22 | | .590 | |
|  | Mouth ***^[S]^*** | .12 | .674 | -.27 | .895 | -.13 | .813 | | -.04 | | .778 | | -.11 | | .634 | |
| Talking | Eyes ***^[S]^*** | .08 | .783 | -.17 | .895 | .12 | .813 | | -.11 | | .584 | | -.09 | | .710 | |
|  | Mouth | .02 | .967 | .02 | .967 | -.01 | .944 | | .13 | | .601 | | .09 | | .678 | |
| **People vs. Geometry** | |  |  |  |  |  |  |  | |  | |  | |  | |  |
| Same-size | People | -.31 | .133 | .01 | .967 | .10 | .639 | | .33 | | .202 | | .14 | | .590 | |
|  | Geometry ***^[S]^*** | **.56*** | **<.050** | -.01 | .947 | -.05 | .881 | | -.26 | | .354 | | -.13 | | .634 | |
| Embedded | People | -.39 | .054 | -.14 | .967 | .15 | .525 | | .13 | | .601 | | .14 | | .590 | |
|  | Geometry ***^[S]^*** | **.48*** | **<.050** | .07 | .895 | -.28 | .813 | | -.15 | | .547 | | -.23 | | .372 | |
| **Biological Motion PLDs** | Upright Figure | .11 | .674 | -.26 | .800 | .11 | .629 | | -.22 | | .403 | | .05 | | .739 | |
|  | Inverted Figure | -.12 | .674 | .01 | .967 | -.30 | .450 | | .09 | | .705 | | -.17 | | .590 | |
| **Referential Attention** | Agent (Face/Hand) | -.12 | .674 | -.21 | .800 | .18 | .508 | | -.08 | | .705 | | -.18 | | .590 | |
|  | Target | -.04 | .928 | .22 | .800 | -.18 | .508 | | .35 | | .202 | | .35 | | .240 | |
|  | Distractors ***^[S]^*** | .30 | .150 | -.06 | .895 | .04 | .881 | | -.10 | | .610 | | -.25 | | .368 | |

Note. All correlations are partial, controlling for % tracking within each stimulus type, with FDR corrected p-values. Pearson’s correlations except [S]=Spearman; ROI=Region of Interest; AOSI=Autism Observation Scale for Infants; MSEL=Mullen Scales of Early Learning; NVDQ=Non-Verbal Developmental Quotient; VABS=Vineland Adaptive Behavior Scales; ABC = Adaptive Behaviour Composite; SS=Standard Score. * FDR corrected *q*<.05.

Table S5. Associations between proportionate gaze data to social vs. non-social ROIs or more vs less socially-salient ROIs and scores on clinical/behavioural phenotyping measures for 47 infants with >30% Tracking Rate.

|  |  | **AOSI** | | **MSEL** | | **VABS** | | | **MSEL** | | | |
| --- | --- | --- | --- | --- | --- | --- | --- | --- | --- | --- | --- | --- |
| **Scene/Trial Type** | **ROI** | **Total *^[S]^*** | *q* | **NVDQ** | *q* | **ABC SS** | *q* | **Receptive AE** | | *q* | **Expressive AE** | *q* |
| **Human Face** |  |  |  |  |  |  |  |  | |  |  |  |
| Blinking | Eyes v. Mouth ***^[S]^*** | .23 | .328 | -.10 | .967 | .13 | .581 | -.13 | | .601 | -.16 | .590 |
| Mouth Moving | Eyes v. Mouth ***^[S]^*** | .32 | .133 | -.12 | .895 | .12 | .813 | -.18 | | .519 | -.05 | .840 |
| Silent/Still (Static) | Eyes v. Mouth | .04 | .928 | -.01 | .967 | .24 | .459 | -.23 | | .403 | -.07 | .678 |
| Mouth Closed (Static) | Eyes v. Mouth | -.01 | .967 | .03 | .967 | .18 | .508 | -.04 | | .790 | .24 | .590 |
| Talking | Eyes v. Mouth ***^[S]^*** | .04 | .928 | -.14 | .895 | .10 | .813 | -.04 | | .778 | .00 | .987 |
| **People vs. Geometry** | |  |  |  |  |  |  |  | |  |  |  |
| Same-size | People v. Geom ***^[S]^*** | **-.45*** | **<.050** | -.07 | .895 | .01 | .958 | .23 | | .354 | .11 | .634 |
| Embedded | People v. Geom ***^[S]^*** | **-.46*** | **<.050** | -.06 | .895 | .23 | .813 | .12 | | .566 | .18 | .538 |
| **Biological Motion PLDs** | Upright v. Inverted | .11 | .674 | -.11 | .967 | .16 | .508 | -.21 | | .403 | .09 | .678 |
| **Referential Attention** |  |  |  |  |  |  |  |  | |  |  |  |
| Target/Person vs Distractors ***^[S]^*** | | -.33 | .133 | -.02 | .947 | .13 | .813 | .17 | | .519 | .29 | .250 |
| Target vs Person/ Distractors ***^[S]^*** | | .01 | .967 | .19 | .895 | -.08 | .829 | .25 | | .354 | .34 | .250 |
| Target vs Distractors ***^[S]^*** | | -.31 | .133 | .03 | .947 | .05 | .881 | .19 | | .519 | .29 | .250 |

Note. All correlations are partial, controlling for % tracking within each stimulus type, with FDR corrected p-values. Pearson’s correlations except [S]=Spearman; ROI=Region of Interest; AOSI=Autism Observation Scale for Infants; MSEL=Mullen Scales of Early Learning; NVDQ=Non-Verbal Developmental Quotient; VABS=Vineland Adaptive Behavior Scales; ABC = Adaptive Behaviour Composite; SS=Standard Score. * FDR corrected *q*<.05.
